# Recruitment to a physical activity intervention study in women at increased risk of breast cancer

**DOI:** 10.1186/1471-2288-9-27

**Published:** 2009-04-27

**Authors:** Larissa A Korde, Amy Micheli, Ashley W Smith, David Venzon, Sheila A Prindiville, Bart Drinkard, Nancy Sebring, Marcia D Smith, Jo Anne Zujewski, Jennifer Eng-Wong

**Affiliations:** 1 Clinical Genetics Branch, NCI, Bethesda MD, USA; 2 Thomas Jefferson University, Philadephia, PA, USA; 3 Division of Cancer Control and Population Sciences, NCI, Bethesda, MD, USA; 4 Center for Cancer Research, NCI, Betehsda, MD, USA; 5 Coordinating Center for Clinical Trials, NCI, Bethesda, MD, USA; 6 NIH Clinical Center, Bethesda, MD, USA; 7 Division of Cancer Treatment and Diagnosis, NCI, Bethesda, MD, USA; 8 Lombardi Comprehensive Cancer Center, Georgetown University Medical Center, Washington, DC, USA

## Abstract

**Background:**

Physical activity is being studied as a breast cancer prevention strategy. Women at risk of breast cancer report interest in lifestyle modification, but recruitment to randomized physical activity intervention studies is challenging.

**Methods:**

We conducted an analysis of recruitment techniques used for a prospective, randomized pilot study of physical activity in women at risk of breast cancer. We evaluated differences in proportion of eligible patients, enrolled patients, and successful patients identified by each individual recruitment method. The Fisher-Freeman-Halton test (an extension of Fisher's exact test from 2 × 2 tables to general row by column tables) was used to compare the success of different recruitment strategies.

**Results:**

We received 352 inquiries from women interested in participating, of whom 171 (54%) were eligible. Ninety-nine women completed a baseline activity evaluation, and 58 (34% of eligible; 16% of total inquiries) were randomized. Recruitment methods fell into three broad categories: media techniques, direct contact with potential participants, and contacts with health care providers. Recruitment strategies differed significantly in their ability to identify eligible women (p = 0.01), and women who subsequently enrolled in the study (p = 0.02).

**Conclusion:**

Recruitment techniques had varying success. Our data illustrate the challenges in recruiting to behavior modification studies, and provide useful information for tailoring future recruitment efforts for lifestyle intervention trials.

**Trial Registration No(s):**

CDR0000393790, NCI-04-C-0276, NCI-NAVY-B05-001

## Background

Breast cancer is the most common malignancy among women in the United States and the second most common cause of cancer mortality [1]. Although tamoxifen and raloxifene have both been shown to decrease breast cancer incidence by approximately 50% [2,3], chemoprevention is not widely accepted by high risk women, mostly due to fear of side effects [4,5]. Consequently, effective and acceptable prevention strategies can have enormous public health implications. Epidemiologic studies suggest that a lifestyle that includes some degree of physical activity is protective against breast cancer. While the amount of activity needed to impart a benefit is unknown, several data from several studies suggest that even a modest amount and intensity of activity is beneficial [6-9]; in the Women's Health Initiative cohort study, brisk walking for 1.25 – 2.5 hours per week resulted in about a 20% decrease in breast cancer risk[7]. Recent data also suggest that physical activity is associated with both decreased breast cancer mortality and reduced risk of recurrence in breast cancer survivors [10,11]. The underlying biologic mechanisms for these effects have yet to be elucidated, but several pathways have been proposed, including alterations of estrogen levels, body weight, growth factor pathways, and immune function [12].

Recruitment to lifestyle modification studies focusing on breast cancer risk reduction is challenging because it requires identifying participants who are not currently exercising, but are, or can be, motivated to participate in a study to increase physical activity. In other exercise intervention studies, only 2 – 20% of potential recruits were successfully randomized [13-16], highlighting the need to identify the most efficacious recruitment strategies. The present recruitment analysis was conducted within a prospective, randomized study of a home-based physical activity intervention comparing the use of a pedometer, an exercise prescription, and a motivational booklet *versus *a control of stretching exercises.

## Methods

We employed various recruitment strategies to identify eligible subjects for a randomized physical activity intervention study in women at increased risk of breast cancer and breast cancer survivors. Simple frequencies were calculated to determine the most efficient method of recruiting eligible subjects. We also evaluated differences in proportion of eligible patients, enrolled patients, and successful patients identified by each individual recruitment technique. Statistical significance was assessed using the Fisher-Freeman-Halton test. The Fisher-Freeman-Halton test is the extension of Fisher's exact test from 2 × 2 tables to general row by column tables. Fisher's exact test gives the probability of observing a table that gives at least as much evidence of association as the one actually observed, given that the null hypothesis is true. All analyses were performed in SAS version 9.1.

### Parent Study Design

The study was conducted at the National Institutes of Health (NIH) Clinical Center, Bethesda MD, and the National Naval Medical Center (NNMC), Bethesda MD. Our goal was to randomize 80 participants to either a physical activity intervention or a control group (stretching exercises). Participants were stratified by risk group (high risk of breast cancer *versus *breast cancer survivors). Inclusion and exclusion criteria are listed in Table 1. Potential participants were pre-screened by phone to assess risk status, self-reported level of physical activity, and potential medical contraindications to regular exercise. Those that met pre-screen criteria were invited to participate in the study, which consisted of a baseline activity evaluation followed by randomization to either intervention or control for those who were eligible. After signing informed consent, each potential enrollee underwent a baseline physical activity evaluation that consisted of wearing a Yamax Digiwalker SW-701 pedometer to wear for one week to objectively determine their current level of activity. For blinding, the pedometer was sealed with a stamped label to prevent the participant from opening it and reading the step count. Initially, we used a mean daily step count of ≤5000 steps/day [17] to define women as sedentary, and therefore eligible for randomization. An interim evaluation in September 2006 revealed that a large percent of women who underwent the baseline evaluation had mean step counts between 5000 and 6000 steps/day, so the protocol was amended to include women who were "low-active," with a baseline mean step count of ≤6000 steps/day. Those meeting the step count criteria were invited to participate in the intervention portion of the study. Participants randomized to the physical activity intervention were asked to gradually increase their daily activity to 10,000 steps/day. The value of 10,000 steps as a physical activity goal can be traced back over thirty years to a slogan used by Japanese walking clubs, and has recently gained popularity in the media and in practice [17]. The goal of 10,000 steps (or an additional 5,000 steps above a sedentary value) roughly corresponds to an additional energy expenditure of 150 kcal or an additional 2 to 2 1/2 miles of walking [18]. This is approximately equal to 30 minutes of activity [17], and therefore satisfies the ACSM-CDC recommendation of 30 minutes of moderately intense physical activity on most days of the week. There is also growing evidence that this level of intervention can have an effect on health related outcomes [19-21].

**Table 1:** Inclusion and exclusion criteria for study participants

Inclusion Criteria
**ALL of the following**:

Age 18–75
Sedentary
Medically fit to exercise
ECOG Performance Status 0–1
If on hormonal therapy, >2 months since starting or changing therapy

**and ANY of the following:**

**Breast cancer survivor:**
History of Stage I, II, or III breast cancer
(> 2 mos since completion of treatment)
**Increased risk of breast cancer:**
Gail model 5 year risk ≥1.67%
Claus model lifetime risk >20%
Previous breast biopsy showing high-risk lesion
(e.g., atypical ductal hyperplasia or lobular neoplasia)
History of appropriately treated ductal carcinoma *in situ*
Known or suspected *BRCA1 *or *BRCA2 *gene mutation

**Exclusion Criteria**

Current or planned pregnancy
Uncontrolled intercurrent illness
Physical conditions that preclude daily walking
(e.g., use of wheelchair, walker, cane, etc)
History of cancer other than breast or non-melanoma skin cancer within 2 years preceding enrollment
Metastatic or recurrent cancer

**Figure 1:**
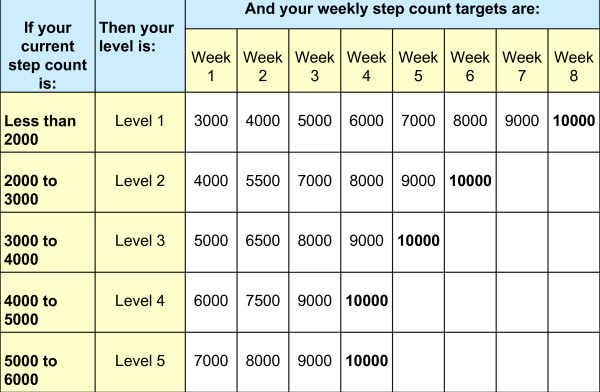
**Exercise Prescription**.

Participants received an educational booklet "Steps to Better Health" (Cooper Institute, Dallas TX) and an exercise prescription (see Figure 1) for incrementally increasing their daily steps over the course of several weeks. This incremental strategy was designed by the study investigators and based on data from successful studies that incentivized incremental increases in walking over the course of 8 weeks [22-24]. Those randomized to the stretching group were given verbal and written instruction on a series of seven standardized exercises designed to improve trunk, hip and shoulder mobility [25], and asked not to alter any other aspect of their physical activities (including walking) during the 12 week study period. All participants were told prior to randomization that they would have the opportunity to participate in the alternate arm at the end of the study. Study personnel contacted all participants by telephone every one to two weeks to assess compliance with the exercise regimens and to provide additional motivation. For study participants not currently meeting their step count goals, study staff followed a telephone script [adapted from the "Steps to Better Health" booklet and the Diabetes Prevention Program Lifestyle Balance Program Materials [26]] addressing a standard list of barriers to exercise and strategies for overcoming them. Study procedures and recruitment materials were reviewed and approved by the Institutional Review Boards of the NIH Clinical Center and the NNMC.

## Results

**Figure 2:**
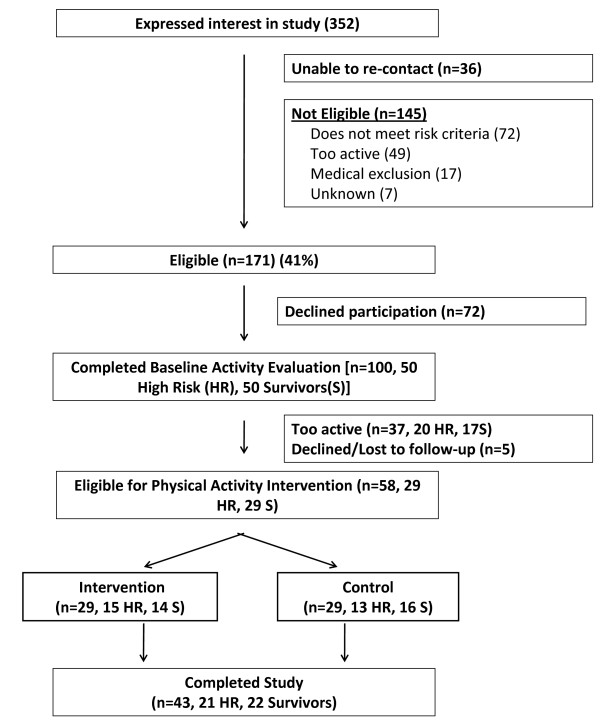
**Summary of Study Enrollment**.

From October 10, 2004 to June 20, 2007, we received 352 inquiries from women interested in participating in the study. Enrollment is summarized in Figure 2. We were able to re-contact 317 women (90%), 171 (54%) of whom were eligible based on the pre-screening criteria. Forty-one percent of those who inquired about the study were not eligible (n = 145); a majority of ineligible patients (n = 72, 50%) did not meet our defined criteria for increased risk of breast cancer or were already exercising regularly (n = 49, 34%). One hundred women (58% of those eligible; 28% of total inquiries) underwent the baseline physical activity evaluation. Of these, 37/100 (37%) were excluded because they were too active on the objective evaluation (i.e., had daily step counts >5,000 or >6,000, as described above) and 5/100 (5%) were excluded for medical reasons; the remaining 58 women (34% of those eligible; 16% of total inquiries) were randomized to either intervention or control.

Recruitment strategies fell into three broad categories: media techniques, direct patient contacts, and contact with health care providers. Specific recruitment efforts and their success rates are shown in Table 2. A majority of inquiries into the study were generated by three sources: a newspaper article describing the study, the recruitment of patients from the NNMC Breast Care Center, and the National Cancer Institute's clinical trials website http://www.cancer.gov. The single most successful method was a news article in the *Gazette*, a local free newspaper delivered to homes in Montgomery County, Maryland. The NCI Press Office alerted the paper's editorial board to the trial, and the editors assigned a reporter to cover the story. This article generated close to one third of total study inquiries, and yielded one-third of total study enrollment. The article was also reasonably efficient in identifying subjects who met eligibility criteria; 56% (60/107) of women who inquired about the study from this source were eligible, *versus *45% (111/245) of those identified through other mechanisms. Of the 60 eligible women identified, 32 (53%) subsequently underwent the baseline PA evaluation, and 13 (22%) were randomized.

**Table 2:** Proportion of study participants identified by individual sources of recruitment

Source	Description	Number of inquiries	Number/% eligible	Number/% enrolled	Number/% randomized	Number/% completed
**Media Techniques**						

*Gazette *article	Article discussing physical activity and breast cancer risk	107	60 (56)	32 (30)	13 (12)	6 (5)
*Express *advertisement	Advertisement in free newspaper in DC metro area	22	5 (23)	1 (5)	1 (5)	1 (5)
TV/print news story	General information in TV/print media	18	7 (39)	4 (22)	4 (22)	4 (22)
NCI Website	Referral service linked to NCI website http://www.cancer.gov	36	19 (53)	14 (39)	8 (22)	8 (22)
Web	Multiple sources, including listservs and posting on group websites	27	10 (37%)	6 (22)	5 (19)	3 (11)

**Direct Patient Contact**						

NCI Study	Recruitment of patients already enrolled in an NCI study	11	10 (91)	8 (73)	4 (36)	3 (27)
NNMC Breast Care Center	Referral of patient at Nat'l Naval Medical Center	45	24 (53)	16 (36)	10 (22)	8 (18)
NNMC Patient mailing	Recruitment letter mailed directly to patients (n = 97)	10	4 (40)	3 (30)	1 (10)	1 (10)
NIH/NNMC employee	Recruitment of current employees of NIH or NNMC	4	1 (25)	1 (25)	1 (25)	1(25)

**Contact with Health Care Providers**						

Physician mailing	Recruitment letter sent to area ob-gyn and oncology practices (n = 500)	16	8 (50)	4 (25)	2 (13)	2 (13)
Community/support group talk	Visit by professional to discuss cancer prevention studies	15	10 (67)	5 (33)	3 (20)	2 (13)

**Unknown**		41	13 (32)	5 (12)	5 (12)	2 (5)

**Total**		**352**	**171**	**99**	**58**	**42**

In an attempt to reach an even broader population, we ran an advertisement in *The Express*, a free publication distributed on the Washington, DC Metrorail system, which has an average weekday ridership of >600,000 people http://wmata.com. The ad ran on three separate occasions over the course of three weeks, at a cost of $1,272, and solicited a total of 22 inquiries. Of these women, 5/22 (23%) women met pre-screen eligibility criteria. One woman underwent the baseline assessment and was subsequently randomized and completed the study.

Recruitment mailings were a reasonably successful means of generating interest in this study, with direct mailings to potential participants having a higher yield than letters to physicians. We sent a letter describing the study to 97 women receiving annual screening and follow-up care at the NNMC Breast Care Center, and received study inquiries from 10 women (10% of those contacted). This was a low cost recruitment method – the only costs were related to labor time for generating the letters and the cost of postage. By contrast, a mailing to local internal medicine and gynecology practices, which was sent to about 500 physicians, at a cost of about $5,000 for the purchase of physician mailing lists, resulted in only 16 referrals to the study.

An important aspect of targeted recruitment is the ability to reach participants who are sufficiently motivated to complete the study. Of 58 participants randomized, 43 (74%) completed the study, yielding an attrition rate of 26% (see Figure 2). Recruiting study participants who had previously participated in clinical trials was most successful for patient retention; 36% (4/11) of previous NIH study participants were randomized, and 75% (3/4) completed the study (3/11, see Table 2). The patterns of retention by source of recruitment (media techniques, direct physician contact, and contact with healthcare providers) approached, but did not reach, statistical significance (p = 0.06).

The difference in success of recruitment methods (media techniques, direct patient contact and contact with healthcare providers) in identifying eligible subjects was statistically significant (p-value for difference in proportion of patients meeting eligibility criteria by individual recruitment strategies = 0.01), suggesting that these differences were not due to chance alone. Similarly, recruitment strategies were significantly different (p = 0.02) in terms of identifying subjects who went on to enroll in the study.

## Discussion

Overall, we were able to recruit and randomize 34% (58/171) of eligible subjects and 16% (58/352) of those who expressed interest. We found that media techniques were most successful for recruiting patients to this lifestyle modification study. The strategies that generated the most patient inquiries were a news article in a local newspaper, the recruitment of patients from the NNMC Breast Care Center, and the NCI clinical trials website. Differences in success of recruitment methods were statistically significant in terms of identifying participants who were eligible and identifying participants who went on to enroll in the study. Differences in retention in the study by recruitment source approached significance.

Our overall recruitment rate of 16%, defined as those who were randomized, was in the range of those reported in other studies [13,14,16], and is similar to that seen for self-referred women in a recent randomized physical activity study for breast cancer survivors [15]. Close to half of the women who expressed interest in participating in our study were not eligible; other investigators have noted similar results [14]. One possible reason is that women tend to overestimate their risk of breast cancer [27], and therefore many women who perceive themselves at high risk do not meet the established study entry criteria. We also had numerous inquiries from women who were already physically active, and had a higher than expected percentage of women who met our pre-screen criteria for being sedentary by self-report, but who were too active for the study when assessed with an objective measure (i.e., a pedometer-determined step count for one week). Increasing the baseline step count cut-off from 5000 to 6000, to include women who were not sedentary by strict criteria, but who could be considered "low-active," partially alleviated this issue. Prior to this modification, 39% of women undergoing the baseline evaluation were too active; after increasing the baseline step count criteria to 6000, only 9% exceeded the baseline activity criteria (data not shown). We initially felt that women with lower baseline step counts would be most likely to benefit from increased physical activity, and would present the greatest opportunity to see measurable changes in both physical activity levels and secondary endpoints. Recently reported studies have allowed higher baseline step counts, and have also shown success in increasing physical activity [16,28]. However, the level of activity needed to impact breast cancer risk, and the incremental benefit of additional activity in already active women, have yet to be determined.

The data provided here can inform the design of future attempts to reach a target population for lifestyle modification studies. An article describing the study that was published in a local newspaper proved to be very successful in generating interest in the study. In contrast, a short newspaper advertisement, which lacked detailed study information, was far less productive. Others have had similar experiences with media techniques [14,29], and data suggest that women who are provided clear, concise information are more likely to participate in research studies than those who feel they are provided a less thorough explanation [30]. In a randomized exercise intervention study for breast cancer survivors, Daley and colleagues had about equal success in generating interest with media techniques and with invitation letters generated by physicians, but media based strategies were more successful in terms of proportion of participants enrolled.

Recruitment mailings have been utilized and evaluated in a number of studies with varying success [14,29,31,32]. In the study described above by Daley and colleagues, in which mailings were particularly effective, eligible women were identified by community oncologists and surgeons, who sent personalized letters to potential study participants[14]. In other studies, a less tailored approach was used; Tworoger and colleagues [32] utilized mailing lists obtained from the Washington State Department of Motor Vehicles, and sent a packet containing an invitation letter, study brochure, eligibility survey and return envelope to over 100,000 women. Seven percent of women responded to the mailing, and 2.5% were enrolled and randomized. In our study, a targeted mailing sent to 97 patients seen in a breast health clinic had a reasonable success rate. This mailing resulted in 10 study inquiries and 4 women subsequently enrolled.

Striking a balance between reaching a large audience and targeting appropriate (and eligible) potential participants is a significant challenge. More broadly based recruitment efforts can reach a larger number of interested participants, but may also create interest among those who do not meet eligibility criteria. The cost of mailings must also be considered, and the more tailored approach may be relatively time intensive.

This study should be interpreted within the context of certain limitations. Recruitment to this study may have been complicated by the fact that women who were interested in a physical activity study were highly motivated to increase their level of physical activity, and therefore potentially less interested in accepting assignment to the control arm of an exercise intervention. Randomizing motivated women to a control arm may discourage participation by those whose original motive was to decrease their risk of breast cancer, an unknown proportion of whom likely assumed that physical activity was going to be effective, despite the absence of proof for this belief. This bias would discourage those most likely to participate. This issue may be alleviated by clear explanation of the opportunity for cross-over. This study did include an informal opportunity for crossover (participants on the stretching arm were offered a pedometer, exercise prescription and educational book at the end of the 3 month study, but were not formally followed for an additional three months). Those randomized to the control arm initially may have felt frustrated with the extra time they must put forth to complete all six months of a study that includes three months of control followed by three months of intervention. By designing the study as a six month cross-over study, all women would have entered the study with the expectation of providing six months of data to the researchers, although with an additional design flaw of asking women to stop exercising.

An additional barrier to the recruitment of eligible subjects is that women who feel compelled to actively reduce their risk of breast cancer are typically more active than those who are not so motivated. One study performed in the context of lifestyle interventions for reduction of diabetes risk suggested that those who are knowledgeable regarding their risk and determined to minimize it are usually already taking preventive measures [33]. By measuring daily step counts at baseline we likely eliminated women who have an active job or who perform daily activities that require more than the routine amount of walking but do not engage in a defined exercise regimen.

## Conclusion

Although we faced multiple challenges, this study was an important first step in launching a lifestyle modification initiative, and our analysis of recruitment and retention addresses the difficulties in mounting this type of clinical trial. In this analysis of our study methods, we have already gained a better understanding of the best way to target future physical activity intervention studies for recruitment.

## Future Research

The identification of practical yet efficacious interventions for increasing physical activity for women at risk of breast cancer and survivors is crucial, as there is mounting evidence that increased physical activity can reap numerous benefits in these populations. Planned analyses of our primary study outcomes will provide important data on the feasibility of a simple, cost-effective, low burden physical activity intervention in breast cancer survivors and women at high risk for the disease. Future studies will be necessary to determine the impact of such an intervention on breast cancer incidence and markers of breast cancer risk.

## Competing interests

The authors declare that they have no competing interests.

## Authors' contributions

LK participated in the study design and coordination, data analysis and drafted the manuscript. AM participated in data collection, analysis and interpretation. AWS participated in the study design. DV participated in the study design and statistical analysis. SAP participated in study design. BD participated in study design and administration. NS participated in study design and administration. MDS participated in study design and administration. JZ and JEW conceived of the study and participated in its design and coordination.

## Pre-publication history

The pre-publication history for this paper can be accessed here:

http://www.biomedcentral.com/1471-2288/9/27/prepub
